# Artificial Intelligence for Glucose Prediction and Glycemic Management in Diabetes: A Systematic Review of Predictive Performance and Clinical Outcomes

**DOI:** 10.7759/cureus.117889

**Published:** 2026-10-13

**Authors:** Mohammed A Alqarni, Abdulmohsen A Alharbi, Salha Q Mashyakhi, Waad S Alamri, Ghala M Asiri, Shahad T Alamri, Shahad Mubarak, Areej N Alsharif, Jamilah A Alshahrani, Albatool Khaled Al Shahrani, Enas S Alqahtani, Bushra A Alsaluli, Saad Dhafer Alshahrani

**Affiliations:** 1 Internal Medicine, King Abdullah Hospital, Asir, SAU; 2 Family Medicine, Aseer Health Cluster, Abha, SAU; 3 General Medicine, King Khalid University, Abha, SAU; 4 General Medicine, University of Bisha, Bisha, SAU; 5 General Surgery, University of Bisha, Bisha, SAU

**Keywords:** artificial intelligence, continuous glucose monitoring, diabetes mellitus, glucose prediction, glycemic management, type 1 diabetes, type 2 diabetes

## Abstract

Artificial intelligence (AI) is increasingly being investigated for glucose prediction and personalized glycemic management in diabetes, particularly through continuous glucose monitoring and multimodal patient data. However, strong predictive performance does not necessarily translate into clinically meaningful benefit. This systematic review evaluated the predictive performance and clinical outcomes of AI-based approaches for glucose prediction, prediction of glycemic events, and AI-enabled glycemic management in adults with diabetes mellitus. A systematic literature search was conducted in PubMed/MEDLINE, Scopus, Web of Science, the Cochrane Library, and Google Scholar from database inception through July 2026. Studies evaluating AI or machine-learning approaches for glucose prediction, prediction of hypoglycemia or hyperglycemia, or AI-enabled glycemic management were included. Data on study characteristics, diabetes type, AI architecture, input variables, prediction targets and horizons, validation strategies, predictive performance measures, clinical outcomes, and methodological quality were extracted. Risk of bias was assessed using design-appropriate validated tools. Thirty studies were included, comprising evidence from type 1 diabetes, type 2 diabetes, and mixed diabetes populations. Most studies investigated short-term glucose forecasting or hypoglycemia prediction, with prediction horizons ranging from approximately 15 minutes to 24 hours. Reported predictive performance was generally promising, although substantial heterogeneity in datasets, model architectures, prediction horizons, outcome definitions, and validation strategies limited direct comparisons. Increasingly, models incorporated insulin, meals, physical activity, demographic characteristics, and other clinical variables to support personalized and multimodal prediction. However, clinical evidence was considerably more limited than technical predictive evidence, with relatively few studies evaluating prospective clinical or decision-support interventions and clinically meaningful outcomes such as time in range or HbA1c. Overall, AI-based approaches demonstrated promising performance for glucose and glycemic event prediction, with a growing emphasis on personalized and multimodal modeling, but evidence for clinical effectiveness remains limited. Stronger prospective, externally validated, and randomized studies using standardized, clinically meaningful outcomes are needed to establish the clinical effectiveness, generalizability, and implementation of AI-based approaches in routine diabetes care.

## Introduction and background

Diabetes mellitus is a major chronic metabolic disorder requiring sustained glycemic management, yet glucose responses vary substantially between individuals [1]. This interindividual variability makes uniform dietary and behavioral recommendations less suitable for many patients and increases the need for individualized approaches to diabetes care. Lifestyle modification remains a central component of diabetes management, but maintaining dietary and behavioral changes can be challenging, creating a need for scalable tools that can support patient self-management and personalized care [2,3]. Artificial intelligence (AI) has therefore emerged as a potential approach for analyzing complex and longitudinal health data and supporting more individualized glycemic management [4,5].

Glycated hemoglobin provides an important measure of longer-term glycemic control but does not fully capture short-term glucose fluctuations or the timing of clinically important glycemic excursions [4,6]. Continuous glucose monitoring (CGM), in contrast, provides repeated measurements of glucose trajectories and can therefore provide a richer representation of daily glycemic patterns. AI models can analyze these longitudinal data and combine them with physical activity, dietary intake, insulin use, and other clinical characteristics to generate individualized predictions [7]. In the Maastricht Study, models based on CGM data predicted glucose at 15 and 60 minutes with root mean square error (RMSE) values of 0.19 and 0.59 mmol/L, respectively, while the addition of accelerometry produced only modest improvement [7].

AI applications extend beyond the prediction of glucose concentration to clinically relevant adverse events. In 440 patients with type 2 diabetes (T2D), machine-learning models were developed for nocturnal hypoglycemia, with the LightGBM model achieving an area under the curve (AUC) of 0.869 [8]. Similarly, deep-learning models have been evaluated across independent populations, demonstrating the importance of external validation and generalizability when translating predictive algorithms into diabetes care [9]. Personalized prediction may further improve the clinical relevance of these systems. Among 67 adults with T2D, individualized models predicted postprandial glucose excursions with a mean F1-score of 75.88%, while the predictors of glycemic excursions differed between individuals [1]. The broader literature also includes substantial evidence from adults with type 1 diabetes (T1D), as well as studies evaluating mixed T1D/T2D populations, indicating that AI-based glucose prediction is being developed across different forms of diabetes.

AI is also increasingly being investigated for direct glycemic management rather than prediction alone [4,10]. Reinforcement-learning approaches have been explored for individualized insulin-regimen optimization in T2D, while prediction-based interventions have attempted to translate anticipated glycemic events into preventive actions. Importantly, an AI-driven digital therapeutic evaluated in 118 adults with non-insulin-treated T2D was associated with a 0.67 percentage-point reduction in HbA1c after three months [2,10]. Other prediction-based interventions have demonstrated the feasibility of linking forecasts to preventive recommendations, although clinical effectiveness cannot be assumed from predictive performance alone [11].

These developments highlight the need to evaluate AI across diabetes populations while considering important differences between T1D, T2D, and mixed populations. In particular, predictive performance demonstrated in one diabetes population may not necessarily be generalizable to another because of differences in treatment patterns, insulin use, glucose dynamics, and clinical context. A systematic assessment should therefore distinguish the underlying diabetes population while examining two related but distinct dimensions: how accurately AI predicts glucose and glycemic events, and whether these predictions translate into meaningful clinical outcomes. Accordingly, this systematic review evaluates the development, validation, and clinical application of AI-based approaches for glucose prediction and glycemic management in adults with diabetes mellitus, encompassing T1D, T2D, and mixed diabetes populations.

## Review

Methods

Study Design

This systematic review was conducted and reported in accordance with the Preferred Reporting Items for Systematic Reviews and Meta-Analyses (PRISMA) 2020 guidelines [12]. The review aimed to identify, critically appraise, and synthesize published evidence on the development, validation, and clinical evaluation of artificial intelligence and machine learning approaches for glucose prediction and AI-enabled glycemic management in adults with diabetes mellitus. The review protocol was not registered in the International Prospective Register of Systematic Reviews (PROSPERO).

The review question was structured using the Population, Intervention, Comparator, and Outcomes (PICO) framework [13]. The population comprised adults with diabetes mellitus, including T1D, T2D, and mixed T1D/T2D populations. The intervention or technology of interest included artificial intelligence, machine learning, deep learning, neural networks, and related predictive algorithms used for glucose forecasting, prediction of hypoglycemia or hyperglycemia, or AI-assisted glycemic management. Comparators included conventional statistical or machine-learning approaches, usual care, standard diabetes management, alternative prediction models, or no comparator, depending on the study design. The primary outcome of interest was glycemic control, particularly time in range (TIR), while secondary outcomes included glucose prediction performance, hypoglycemia and hyperglycemia prediction, time below or above range, HbA1c, glycemic variability, and other clinically relevant glycemic outcomes.

Search Strategy

A comprehensive systematic literature search was performed from database inception through July 2026. The search strategy was developed to identify studies combining concepts related to diabetes mellitus, artificial intelligence or machine learning, glucose prediction or glycemic management, and clinically relevant glycemic outcomes. Both controlled vocabulary and free-text terms were combined using Boolean operators and truncation where appropriate. Search strategies were adapted to the syntax and indexing requirements of each database.

No restrictions on publication year were applied during the search. The principal electronic databases searched were PubMed, Scopus, and Web of Science. Additional sources were searched where applicable to identify potentially relevant publications. The complete database-specific search strategies are provided in Appendix 1.

Information Sources

Electronic searches were conducted in PubMed/MEDLINE, Scopus, and Web of Science Core Collection. Additional searching was performed through the Cochrane Library and Google Scholar. Reference lists of eligible studies were also examined to identify potentially relevant studies not retrieved through the electronic database searches.

Eligibility Criteria

Studies were eligible for inclusion if they met the following criteria: (1) included human adults with diabetes mellitus, including T1D, T2D, or mixed diabetes populations; (2) developed, validated, evaluated, or clinically implemented an artificial intelligence- or machine learning-based approach for glucose prediction or glycemic management; (3) used glucose-related information, particularly CGM data, either alone or in combination with insulin, dietary, physical activity, physiological, demographic, or other clinical variables; and (4) reported at least one relevant glycemic outcome, including glucose prediction performance, hypoglycemia or hyperglycemia prediction, TIR, time below range, time above range, HbA1c, glycemic variability, or another clinically relevant measure of glycemic control.

Studies involving mixed diabetes populations were eligible when participants with diabetes were included in the evaluated population and the reported analysis was relevant to glucose prediction or glycemic management in people with diabetes. Where diabetes-specific results were available, these were extracted and reported according to diabetes type. Diabetes type was therefore recorded for each included study and considered when interpreting the findings.

Studies evaluating AI models for short-term glucose forecasting or prediction of glycemic events were eligible even when clinical intervention outcomes were not reported, because characterization of predictive performance constituted an important component of the review objective. Studies evaluating AI-generated recommendations or decision-support interventions were also eligible when they assessed subsequent glycemic outcomes.

Studies were excluded if they (1) involved populations without diabetes mellitus and did not provide relevant data from participants with diabetes; (2) did not use an artificial intelligence, machine learning, deep learning, or predictive algorithmic approach; (3) did not address glucose prediction, glycemic-event prediction, or AI-assisted glycemic management; (4) did not report an eligible glycemic outcome; (5) were reviews, systematic reviews, meta-analyses, protocols, editorials, letters, conference abstracts, or other non-original publications; or (6) lacked sufficient information to determine the relevance of the AI approach or its glycemic outcomes.

Study Selection and Screening

All retrieved records were imported into Rayyan, a web-based platform for systematic review screening [14]. Duplicate records were identified and removed before screening. Title and abstract screening was conducted using the predefined eligibility criteria, followed by full-text assessment of potentially eligible reports. Studies were retained for full-text review when the title and abstract indicated a potential relationship with AI-based glucose prediction, glycemic-event prediction, or AI-enabled glycemic management. Full texts were subsequently assessed to confirm eligibility. Reasons for exclusion at the full-text stage were documented. Where multiple publications appeared to originate from the same dataset, the reports were examined for overlap, and the most relevant or informative report was retained where appropriate.

Data Extraction

Data were extracted using a standardized data extraction framework developed specifically for this review. Extracted information included author, publication year, country, study design, population characteristics, sample size, diabetes type, data source, CGM characteristics, prediction target, prediction horizon, AI or machine learning architecture, input variables, model development strategy, validation approach, comparator models, and reported outcomes.

Data extraction was independently performed or verified by two reviewers using the standardized extraction framework. Discrepancies were resolved through discussion and, when consensus could not be reached, adjudicated by a third reviewer. Particular attention was given to verification of diabetes type, sample size, AI architecture, prediction target and horizon, validation strategy, and reported predictive performance measures because of the substantial technical heterogeneity across studies.

For prediction-model studies, model performance measures such as area under the receiver operating characteristic curve (AUROC), area under the precision-recall curve (AUPRC), sensitivity, specificity, accuracy, F1-score, mean absolute error, root mean squared error, mean absolute relative difference, calibration measures, and other reported performance metrics were extracted where available. For intervention studies, information on the intervention, comparator, follow-up period, TIR, time below range, time above range, HbA1c, hypoglycemic events, and other clinical outcomes was extracted. Detailed AI architecture and model characteristics were summarized separately, including model type, input modalities, prediction horizon, validation strategy, and external validation where reported.

Risk-of-Bias Assessment

Risk of bias was assessed using a design-specific approach. Prediction-model development and evaluation studies were assessed using PROBAST+AI, the updated Prediction Model Risk-of-Bias Assessment Tool incorporating artificial intelligence and machine-learning considerations [15]. PROBAST+AI evaluates risk of bias across four domains: participants and data sources, predictors, outcome, and analysis, and assesses applicability across the relevant domains.

Randomized clinical intervention studies were assessed using the Cochrane Risk of Bias 2 (RoB 2) tool [16]. RoB 2 evaluates five domains: bias arising from the randomization process, bias due to deviations from intended interventions, bias due to missing outcome data, bias in measurement of the outcome, and bias in selection of the reported result.

Nonrandomized studies evaluating the effects of an AI-driven intervention were assessed using ROBINS-I (Risk of Bias In Non-randomized Studies of Interventions) [17], which evaluates seven domains: bias due to confounding, selection of participants, classification of interventions, deviations from intended interventions, missing data, measurement of outcomes, and selection of the reported result. Risk-of-bias assessments were performed according to the design and primary objective of each study. The detailed assessments are presented in the Appendices.

Data Synthesis

Because of substantial methodological and clinical heterogeneity across the included studies, findings were primarily synthesized narratively and descriptively. Heterogeneity was observed in AI architectures, data sources, CGM devices, predictor variables, prediction horizons, outcome definitions, validation strategies, and performance metrics, which limited the validity of pooling predictive performance measures across studies. The synthesis therefore focused on identifying patterns in AI applications, model characteristics, predictive performance, validation strategies, and clinical outcomes. Diabetes type was considered when interpreting the evidence, and findings from T1D, T2D, and mixed populations were distinguished where relevant. Studies were considered according to major AI applications, including glucose forecasting, hypoglycemia prediction, hyperglycemia prediction, postprandial or meal-related glucose prediction, personalized or multimodal prediction, and AI-enabled glycemic decision support or preventive intervention.

Application categories were assigned using predefined criteria based on the primary prediction target and intended clinical use reported in each study. Glucose forecasting referred to the prediction of future glucose concentrations; hypoglycemia prediction referred to the prediction of low-glucose or hypoglycemic events; hyperglycemia prediction referred to the prediction of above-range glucose or hyperglycemic events; postprandial or meal-related prediction referred to the prediction of glucose excursions associated with meals or dietary intake; personalized or multimodal prediction referred to patient-specific models or models integrating multiple data modalities beyond glucose measurements alone; and AI-enabled decision support or preventive intervention referred to approaches in which predictions were linked to clinical, behavioral, or preventive recommendations or interventions. A single study could contribute to more than one application category when it evaluated multiple distinct applications. Accordingly, the counts reported for application categories represent study applications rather than mutually exclusive independent studies.

Emphasis was placed on the relationship between technical model performance and clinically meaningful outcomes, especially TIR, time below range, time above range, HbA1c, hypoglycemic events, and glycemic variability. Predictive performance and clinical effectiveness were interpreted separately to avoid equating statistical model accuracy with demonstrated patient benefit. Given the substantial heterogeneity and the limited number of studies reporting sufficiently comparable clinical outcomes, no quantitative meta-analysis was performed, and the evidence was synthesized using structured narrative methods and descriptive tables. Predictive performance ranges were treated as descriptive summaries of reported study-level results and were not interpreted as pooled estimates.

Results

Study Selection

The database search identified 1,769 records, comprising 356 from PubMed, 834 from Scopus, 447 from Web of Science, 82 from the Cochrane Library, and 50 from Google Scholar. After removal of 370 duplicate records, 1,399 records underwent title and abstract screening. Of these, 1,365 records were excluded because they did not address diabetes or glycemic management (n = 480), did not use artificial intelligence, machine learning, or predictive algorithms (n = 310), did not evaluate glycemic prediction, glucose forecasting, or AI-driven glycemic management (n = 240), involved an ineligible population (n = 170), were non-original or otherwise ineligible study designs (n = 105), or did not report an eligible glycemic outcome (n = 60). Thirty-four reports were assessed at the full-text stage, with four excluded because of an ineligible AI/ML prediction or intervention design (n = 1), an ineligible population or absence of relevant diabetes-specific data (n = 1), absence of an eligible glycemic outcome (n = 1), or duplicate or overlapping data (n = 1). Thirty studies were ultimately included in the systematic review [1,2,7-9,11,17-40]. The complete selection process is presented in Figure 1. 

**Figure 1:**
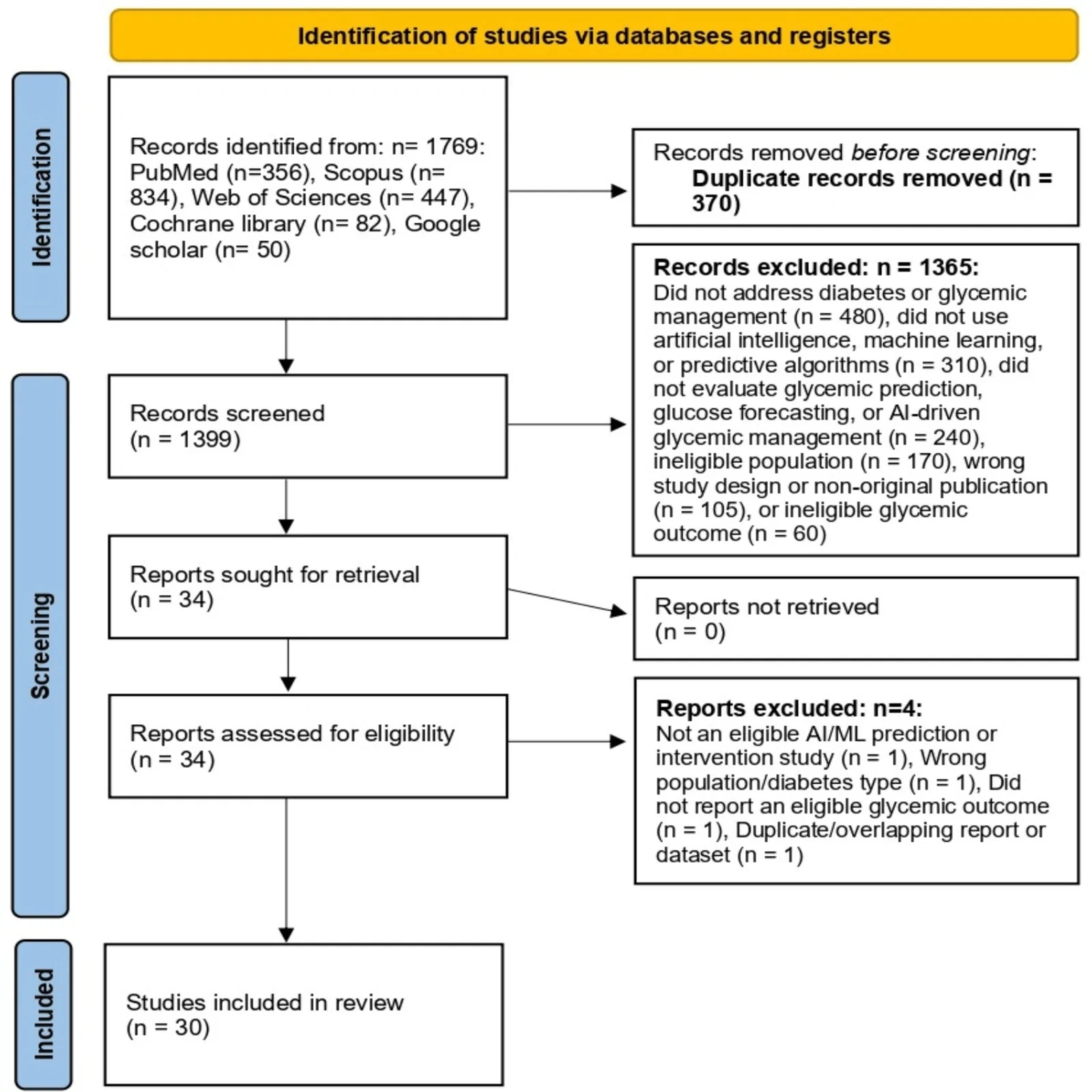
PRISMA 2020 flow diagram of the study selection process PRISMA: Preferred Reporting Items for Systematic Reviews and Meta-Analyses.

Characteristics of Included Studies

The 30 included studies represented a heterogeneous body of evidence spanning adults with T1D, T2D, and mixed diabetes populations. The studies evaluated AI-based glucose forecasting, prediction of adverse glycemic events, personalized prediction, and AI-enabled clinical or behavioral decision support. Most studies were conducted in T1D populations, whereas a smaller number specifically evaluated T2D, with several studies including mixed T1D/T2D populations. Study characteristics, populations, diabetes type, sample sizes, prediction targets, prediction horizons, and study designs are summarized in Table 1. 

**Table 1:** Characteristics of studies evaluating artificial intelligence-driven predictive glucose monitoring and decision support in adults with diabetes For studies including broader populations, only data relevant to participants with diabetes mellitus were considered for this review where diabetes-specific results were available. CGM, continuous glucose monitoring; MDI, multiple daily injections; T1D, type 1 diabetes; T2D, type 2 diabetes; IoMT, Internet of Medical Things; LLM, large language model; NR, not reported.

Study	Study design/setting	Diabetes population	Sample size	Age	Insulin treatment	CGM/data source	Additional data
Alkhateeb et al., 2026 [18]	Prediction-model development/validation; free-living Ramadan cohort	T1D	33	Adults	NR	CGM	Wrist-worn wearable/behavioral data
Alvarado et al., 2023 [19]	Prediction-model study	T1D	30	Adults	NR	FreeStyle Libre CGM	NR
Amar et al., 2020 [20]	Prediction-model development/validation	T1D	141 real-world patients	Adult and age-stratified analyses	NR	CGM; >1.5 million measurements	Simulated CGM data
Brügger et al., 2025 [1]	Prediction-model study	T2D	67	Adults	NR	CGM; ShanghaiT2DM	Postprandial/meal data
Cichosz et al., 2021 [21]	Prediction-model development/validation	T1D	225	Mean 44 years	NR	CGM; ~315,000 h	NR
Cichosz et al., 2021 [22]	Neural-network development and external validation	T1D	278 in external validation	Adults	NR	CGM	NR
Cichosz et al., 2024 [23]	Prediction-model development + external validation	T1D	205 discovery; 149 CITY; 104 REPLACE-BG	Adults	NR	CGM	Clinical/demographic predictors
Gong et al., 2024 [8]	Prediction-model development/validation	T2D	440	Adults	NR	CGM; ≥24 h	Clinical variables
Herrero et al., 2024 [24]	Predictive application/model validation	T1D and T2D	21 T1D MDI; 59 T2D MDI; 226 T1D pump	Adults	MDI/pump	CGM	NR
Kannenberg et al., 2026 [2]	Digital therapeutic intervention	Non-insulin-treated T2D	118	Adults	No insulin	CGM	Lifestyle, meals and behavioral data
Khadem et al., 2026 [25]	Prediction-model development/validation	T1D	NR	Adults	NR	OhioT1DM CGM	NR
Kim et al., 2022 [26]	Prediction-model study	T2D	29 for multivariate modeling	Adults/hospitalized patients	Insulin data available	CGM	Carbohydrate and insulin data
Kladov et al., 2024 [27]	Prediction-model development/validation	T1D	570	Adults	NR	Real-time CGM	NR
Kozinetz et al., 2024 [28]	Prediction-model development/validation	T1D	380	Adults	MDI	CGM	NR
Kushner et al., 2020 [29]	Patient-specific prediction-model study	T1D	24	Adults	NR	CGM	Insulin and carbohydrate data
Lara-Abelenda et al., 2025 [30]	Personalized prediction-model development/validation	T1D	T1DEXI cohort	Adults	MDI and pump	CGM	NR
Luo et al., 2025 [31]	Foundation-model development/validation	T1D and T2D	592	Adults	NR	~1.6 million CGM records	NR
Mosquera-Lopez et al., 2019 [32]	Prediction-model development and clinical testing	T1D	10 clinical test participants; large training dataset	Adults	NR	CGM	Insulin data
Mosquera-Lopez and Jacobs, 2022 [33]	Prediction-model development/validation	T1D	175 developments; 75 test	Adults	NR	CGM	Insulin data
Mosquera-Lopez et al., 2023 [34]	Prediction + intervention/decision-support development	T1D	366	Adults	MDI	CGM	Physical activity data
Mosquera-Lopez et al., 2025 [11]	Randomized crossover clinical trial	T1D	20	Adults	MDI	CGM	Bedtime Smart Snack application
Parcerisas et al., 2022 [35]	Prediction/intervention simulation study	T1D	NR	Adults	MDI	CGM	Physical activity data
Rabby et al., 2021 [36]	Prediction-model development/validation	T1D	6	Adults	NR	OhioT1DM CGM	Meals, insulin and steps
Rodríguez-Almeida et al., 2025 [37]	Personalized prediction-model development/validation	T1D	29	Adults	NR	CGM; 1 year	NR
Shao et al., 2024 [9]	Prediction-model development + external validation	T1D and T2D	192 developments; 427 validation	Adults	NR	Multiple CGM datasets	NR
Shilo et al., 2022 [38]	Personalized prediction-model study	T1D	121; 75 adults	Adults and younger participants	Insulin pump	CGM	Meals, clinical and microbial data
van Doorn et al., 2021 [7]	Prediction-model study; Maastricht Study	Normal glucose regulation, prediabetes and T2D	851	40–75 years	NR	CGM	Physical activity/accelerometry
Zhu et al., 2022 [39]	Wearable/decision-support model development	T1D	~12	Adults	NR	CGM	Wearable sensor data
Zhu et al., 2023 [40]	IoMT/model development and validation	T1D	47 clinical subjects	Adults	NR	CGM	Wearable/IoMT data
Zhu et al., 2026 [41]	Multimodal LLM development and external validation	T1D; external T2D/non-diabetes	226 developments; 207 external	Adults	NR	CGM	Activity + EHR data

The methodological approaches varied substantially. Models included recurrent neural networks, long short-term memory (LSTM) networks, convolutional and attention-based architectures, transformer-based models, random forests, support-vector methods, gradient-boosting approaches, and other machine-learning algorithms. Most prediction models used CGM data, either alone or combined with insulin administration, carbohydrate intake, physical activity, demographic characteristics, or other clinical variables. Detailed AI architectures, input features, validation approaches, and model characteristics are presented in Table 2.

**Table 2:** Characteristics of artificial intelligence models, prediction targets, and intended clinical applications AI, artificial intelligence; CNN, convolutional neural network; CGM, continuous glucose monitoring; GBT, gradient-boosted trees; GRU, gated recurrent unit; LLM, large language model; LSTM, long short-term memory; ML, machine learning; RF, random forest; RNN, recurrent neural network; SVM, support vector machine.

Study	AI/ML approach	Input data	Prediction target	Prediction horizon	Personalized model	Intended application
Alkhateeb et al., 2026 [18]	Multimodal recurrent deep learning	CGM + wearable/behavioral features	Hypoglycemia	1 h	Yes/behavior-aware	Early warning
Alvarado et al., 2023 [19]	Wavelet transform + CNN/DenseNet	CGM	Hypoglycemia	Up to 24 h	No/NR	Predictive alert
Amar et al., 2020 [20]	ANN and gradually connected neural network; tree-based models	CGM	Future glucose	30/60 min	NR	Glucose forecasting
Brügger et al., 2025 [1]	XGBoost + SHAP	CGM + meal/postprandial data	Postprandial glucose excursion	Postprandial	Yes	Personalized dietary decision support
Cichosz et al., 2021 [21]	LSBoost, bagging, neural networks, penalty-weighted ML	CGM	Future glucose	10–60 min	NR	Glucose forecasting
Cichosz et al., 2021 [22]	Neural-network regression	CGM	Future CGM glucose	15 min	NR	Glucose forecasting
Cichosz et al., 2024 [23]	XGBoost	CGM + clinical predictors	Excessive hypoglycemia risk	1 week	NR	Risk stratification
Gong et al., 2024 [8]	Logistic regression, RF, LightGBM	CGM + clinical variables	Nocturnal hypoglycemia	Overnight	NR	Predictive alert/risk assessment
Herrero et al., 2024 [24]	ML predictive models	CGM	Future glucose + low-glucose risk	30–120 min	NR	Predictive CGM application
Kannenberg et al., 2026 [2]	Proprietary AI/predictive algorithm	CGM + lifestyle/meal data	Personalized glycemic response	Ongoing/repeated	Yes	Lifestyle intervention/digital therapeutic
Khadem et al., 2026 [25]	Collaborative/adversarial deep learning	CGM	Future glucose	30/60 min	Yes	Personalized forecasting
Kim et al., 2022 [26]	RNN, LSTM, GRU, BiLSTM + genetic algorithm ensemble	CGM + insulin + carbohydrate	Future glucose	15/30/60 min	NR	Glucose forecasting
Kladov et al., 2024 [27]	Clustering + GBT/RF	CGM	Nocturnal glucose/low glucose	15/30 min	Subgroup-specific	Predictive monitoring
Kozinetz et al., 2024 [28]	MLP, CNN, RF, GBT	CGM	Nocturnal target/high/low glucose	30 min	NR	Predictive alert
Kushner et al., 2020 [29]	Patient-specific shallow neural networks + transfer learning	CGM + insulin + carbohydrate	Future glucose	60–240 min	Yes	Advisory/closed-loop support
Lara-Abelenda et al., 2025 [30]	Transformer/LLM-based personalized forecasting	CGM	Future glucose	60/90/120 min	Yes	Personalized glucose forecasting
Luo et al., 2025 [31]	Transformer-based CGM foundation model	CGM	Future glucose	Up to 2 h	Transfer/zero-shot	Generalizable glucose forecasting
Mosquera-Lopez et al., 2019 [32]	LSTM + patient-specific smoothing	CGM + insulin	Glucose, hypo-/hyperglycemia	~30 min	Yes	Predictive alerts
Mosquera-Lopez and Jacobs, 2022 [33]	LSTM	CGM + insulin	Future glucose	30/60 min	Partly personalized	Glucose forecasting
Mosquera-Lopez et al., 2023 [34]	Evidential neural network	CGM + physical activity	Nocturnal hypoglycemia	0–4/4–8 h	Yes	Predictive decision support
Mosquera-Lopez et al., 2025 [11]	Evidential neural network + Smart Snack algorithm	CGM	Nocturnal hypoglycemia	Overnight	Yes	Personalized preventive intervention
Parcerisas et al., 2022 [35]	ML prediction + personalized/population models	CGM + physical activity	Nocturnal hypoglycemia	Overnight	Yes	Preventive carbohydrate recommendation
Rabby et al., 2021 [36]	Stacked LSTM + Kalman smoothing	CGM + meals + insulin + steps	Future glucose	30/60 min	NR	Glucose forecasting
Rodríguez-Almeida et al., 2025 [37]	Fully personalized deep learning	CGM	Future interstitial glucose	Up to 1 h	Yes	Personalized predictive monitoring
Shao et al., 2024 [9]	LSTM; SVM/RF comparators	CGM	Hypoglycemia	30 min	NR	Predictive alert
Shilo et al., 2022 [38]	Personalized ML model	CGM + insulin + meals + clinical/microbial data	Postprandial glucose response	Post-meal	Yes	Personalized meal/insulin decisions
van Doorn et al., 2021 [7]	Machine-learning models	CGM + physical activity	Future glucose	15/60 min	NR	Glucose forecasting
Zhu et al., 2022 [39]	Deep learning	CGM + wearable data	Glucose, hypo-/hyperglycemia	Short-term	NR	Smartphone decision support
Zhu et al., 2022 [40]	Attention-based evidential recurrent neural network	CGM + IoMT/wearable data	Future glucose + hypoglycemia	Short-term	NR	Real-time predictive management
Zhu et al., 2026 [41]	Multimodal on-device LLM (LLaMA-based)	CGM + activity + EHR	Future glucose + hypoglycemia	30 min and longer	NR	Real-time decision support

AI Applications and Predictive Performance

The evidence was dominated by short-term glucose forecasting and prediction of hypoglycemia, generally using prediction horizons ranging from approximately 15 to 60 minutes. Across studies, AI models generally demonstrated acceptable to high predictive performance, although direct comparison was limited by substantial variation in datasets, prediction horizons, outcome definitions, validation strategies, and performance metrics. For example, van Doorn et al. evaluated multiple statistical and machine-learning architectures in 851 individuals and found that a recurrent neural network performed best at 15 minutes, whereas LSTM achieved the best performance at 60 minutes [7].

The evidence base was predominantly derived from T1D populations, while fewer studies specifically evaluated T2D or mixed T1D/T2D populations. Therefore, reported predictive performance should be interpreted in the context of diabetes type and the underlying treatment and data collection setting.

Hypoglycemia prediction constituted one of the most frequently investigated applications. Models commonly used recent CGM trajectories to generate early warnings of impending hypoglycemia, with increasingly sophisticated approaches incorporating insulin, meal, and contextual information. Studies also increasingly investigated hyperglycemia prediction, although this evidence base was smaller. Personalized and multimodal models generally demonstrated competitive or improved predictive performance when CGM was combined with insulin, dietary, physical activity, or individual-level characteristics. The principal findings across predefined AI application categories are summarized in Table 3. Studies were classified according to their primary prediction target and intended clinical use; because individual studies could address more than one prediction target or application, categories were not mutually exclusive.

**Table 3:** Aggregated predictive performance and clinical outcomes according to AI application The numbers represent study applications rather than mutually exclusive studies; therefore, a single study may contribute to more than one category, and the category counts should not be summed to obtain the total number of included studies. RMSE values are expressed in mg/dL where reported. AUROC: area under the receiver operating characteristic curve; CGM: continuous glucose monitoring; HbA1c: glycated hemoglobin; TIR: time in range; TAR: time above range.

AI application	No. of study applications	Main prediction target	Typical prediction horizon	Summary of predictive performance	Clinical/glycemic outcomes	Overall interpretation
Short-term glucose forecasting	14	Future glucose concentration	15–120 min	Most models demonstrated good short-term prediction, with reported RMSE generally ranging from approximately 6–32 mg/dL at shorter horizons. Prediction error generally increased as the forecasting horizon increased.	Direct evidence of improved clinical outcomes was limited because most studies evaluated model performance rather than clinical implementation.	Strong technical evidence; limited evidence for clinical benefit
Hypoglycemia prediction	9	Future hypoglycemia/low-glucose events	15 min–24 h	Reported discrimination was generally promising, with AUROC values approximately 0.71–0.98 in studies reporting AUROC. Sensitivity and specificity varied according to outcome definition and prediction horizon.	Several studies used predictions to trigger preventive actions, including carbohydrate recommendations. Evidence for reduction in hypoglycemia was promising but heterogeneous.	Promising predictive performance; limited clinical validation
Nocturnal hypoglycemia prediction	5	Overnight hypoglycemia	30 min–overnight	Models demonstrated moderate-to-high discrimination, with reported AUROC values around 0.71–0.87 in major validation studies.	Personalized preventive strategies were associated with reductions in predicted or observed nocturnal hypoglycemia in selected studies.	Clinically promising but supported by relatively few studies
Postprandial/meal-response prediction	3	Postprandial glucose excursion or individualized glycemic response	Post-meal/1–3 h	Personalized models demonstrated useful prediction of interindividual glycemic responses, with performance varying according to meal composition, baseline glucose and additional behavioral predictors.	Potential to support individualized dietary and meal-related recommendations; direct clinical outcome evidence remains limited.	Emerging evidence for personalized diabetes management
Hyperglycemia prediction	3	Future hyperglycemia/above-range glucose	30–120 min	Predictive performance was generally acceptable, although substantially fewer studies specifically focused on hyperglycemia than on glucose forecasting or hypoglycemia.	Direct evidence that hyperglycemia prediction improves TIR or reduces TAR was limited.	Limited but promising evidence
Personalized/multimodal glucose prediction	7	Future glucose or glycemic events using patient-specific and multimodal data	30 min–several hours	Personalized and multimodal approaches generally showed improved or competitive performance compared with conventional models, particularly when CGM was combined with insulin, meals, physical activity, or clinical data.	Clinical implementation remains limited; evidence suggests potential for individualized decision support rather than established clinical effectiveness.	Rapidly emerging field with limited clinical validation
AI-driven decision support/preventive intervention	4	Prevention or management of adverse glycemic events	Real-time/overnight/longitudinal	Prediction models were generally sufficiently accurate to support alerts or individualized recommendations, but performance was not always the primary endpoint.	Clinical studies demonstrated potential improvements in HbA1c, reduction in nocturnal hypoglycemia, and simulated/observed improvements in glycemic control. Evidence for TIR improvement remains limited.	Smallest evidence base but greatest clinical relevance

Clinical and Glycemic Outcomes

Evidence for clinical effectiveness was substantially more limited than evidence for predictive performance. Only a small subset of studies evaluated AI-generated predictions within a clinical or decision-support context, and outcome definitions varied considerably. Direct evidence for improvement in TIR was particularly limited, preventing meaningful quantitative synthesis across studies. Overall, the available clinical evidence suggested potential improvements in glycemic control, reductions in hypoglycemia, and improvements in HbA1c; however, these findings were based on a small and heterogeneous subset of studies and should not be interpreted as evidence of established clinical effectiveness.

Among the included studies, the clearest real-world intervention evidence came from Kannenberg et al., who evaluated an AI-driven CGM-based digital therapeutic in 118 adults with non-insulin-treated type 2 diabetes. After three months, HbA1c decreased by 0.67 percentage points overall and by 1.08 percentage points among participants with baseline HbA1c ≥7%; the proportion achieving HbA1c <7% increased from 38% to 60%. Other studies evaluated personalized recommendations or simulated decision-support strategies, but these generally reported predicted or simulated benefits rather than prospectively observed improvements in TIR.

Risk of Bias

Risk of bias was assessed according to study design using PROBAST+AI for prediction-model studies, RoB 2 for the randomized clinical intervention, and ROBINS-I for the nonrandomized intervention-effect study. Detailed assessments are provided in the supplementary risk-of-bias tables. Prediction-model studies showed substantial heterogeneity in methodological quality, with the principal concerns relating to participant selection, sample size, validation strategies, calibration, and analysis. External validation was less consistently reported than internal validation.

The randomized intervention study was judged to have some concerns overall, primarily because of limited reporting of the randomization process, while the remaining RoB 2 domains were judged to be at low risk. The nonrandomized AI-driven intervention study was judged to have a serious overall risk of bias, driven mainly by potential confounding inherent to the pre-post design. Overall, the risk-of-bias assessment indicates that the promising predictive performance of AI models should be interpreted separately from the considerably more limited evidence for causal clinical effectiveness.

Discussion

This systematic review synthesized evidence from 30 studies evaluating AI and machine-learning approaches for glucose prediction and AI-enabled glycemic management in adults with diabetes mellitus. Overall, the literature demonstrates substantial progress from conventional short-horizon glucose forecasting toward personalized, multimodal, and potentially clinically actionable prediction systems. However, the evidence also reveals an important translational gap: predictive performance has advanced considerably faster than evidence demonstrating improvements in patient-centered glycemic outcomes. The evidence base was predominantly derived from T1D populations, with fewer studies specifically evaluating T2D or mixed T1D/T2D populations. This distribution is important when interpreting the generalizability of reported performance because treatment regimens, insulin exposure, behavioral patterns, glucose dynamics, and CGM use may differ substantially across diabetes populations.

The most consistent application of AI was short-term glucose forecasting, particularly the prediction of impending hypoglycemia and hyperglycemia. Models increasingly use longitudinal CGM trajectories, often supplemented by insulin, physical activity, meal-related, physiological, and contextual information. Cichosz et al. demonstrated that more advanced machine-learning models could improve glucose prediction compared with simpler approaches, particularly at clinically relevant prediction horizons [21]. Their work illustrates the fundamental advantage of AI in capturing nonlinear temporal relationships within dense CGM data. Similarly, Mosquera-Lopez and Jacobs showed that an LSTM model could predict glucose up to 60 minutes ahead and achieved RMSE values of approximately 19.8 mg/dL at 30 minutes and 33.2 mg/dL at 60 minutes [33].

Nevertheless, greater algorithmic complexity did not consistently translate into large improvements over conventional methods. Mosquera-Lopez and Jacobs reported that the performance difference between LSTM, Ridge regression, and random forest was less than 2 mg/dL in RMSE [33]. This finding is important because it challenges the assumption that deep-learning architecture alone determines clinical superiority. The value of a sophisticated AI model should instead be judged according to whether it provides meaningful improvements in prediction, generalizability, calibration, personalization, or downstream clinical outcomes.

A major theme across the literature was the prediction of clinically important glycemic excursions, particularly hypoglycemia. Gong et al. developed machine-learning models for nocturnal hypoglycemia and demonstrated strong discrimination, with the best-performing LightGBM model achieving an AUC of approximately 0.87 [8]. This supports the concept that AI can integrate historical glucose patterns with clinical and treatment-related characteristics to identify patients at increased short-term risk.

The literature also shows increasing sophistication in incorporating contextual information. Alkhateeb et al. developed a multimodal model integrating CGM with wearable-derived physiological, behavioral, and circadian information for hour-ahead hypoglycemia prediction during Ramadan fasting [18]. The model maintained strong performance across Ramadan and post-fasting conditions, with the bidirectional LSTM (BiLSTM) achieving ROC-AUC values of 0.917 and 0.911 in cross-phase validation [18]. This is particularly relevant because glycemic dynamics are strongly influenced by behavior, meal timing, sleep, and physical activity. Incorporating these variables may therefore improve prediction beyond glucose-only models.

Similarly, Mosquera-Lopez et al. developed an evidential neural network for predicting nocturnal hypoglycemia at different periods of the night [34]. The model generated separate risk estimates according to the expected timing of the event, providing information that could potentially be translated into individualized preventive interventions. The progression from simply predicting glucose concentration to predicting when and why clinically relevant excursions are likely to occur represents an important step toward actionable AI.

Another major development is the movement from population-level models toward individualized prediction. Kushner et al. used patient-specific neural networks and transfer learning to forecast future glucose over longer horizons [29], while Lara-Abelenda et al. investigated personalized transformer-based approaches across prediction horizons extending to 120 minutes [30]. These approaches recognize that glucose dynamics differ substantially between individuals because of differences in insulin sensitivity, treatment regimens, dietary patterns, physical activity, and baseline glycemic variability.

The importance of personalization is also supported by Mosquera-Lopez and Jacobs, who demonstrated that prediction accuracy was related to individual glucose variability and explored clustering approaches to identify groups for which customized LSTM models might provide better performance [33]. Thus, future AI systems may benefit from hybrid architectures that combine population-level learning with patient-specific adaptation rather than relying exclusively on a single model trained across heterogeneous populations.

Foundation-model and large-model approaches represent a newer stage of this evolution. Luo et al. evaluated a CGM foundation model capable of forecasting glucose across different populations and prediction horizons [31], while Zhu et al. investigated a large language model-based glucose forecasting framework using an adaptor architecture with multiple time-series baselines [41]. These approaches may eventually improve transferability between datasets and populations, but their clinical value will depend on rigorous external validation rather than performance on isolated benchmark datasets.

Diabetes type and generalizability are particularly important considerations when interpreting these findings. A substantial proportion of the prediction-model literature was conducted in T1D populations, whereas fewer studies specifically evaluated T2D. Although the underlying prediction tasks may appear similar, the clinical contexts are not necessarily interchangeable. Differences in insulin dependence, medication exposure, meal patterns, physical activity, glucose variability, and CGM utilization may influence both model inputs and prediction targets. Accordingly, strong performance in a T1D cohort should not automatically be assumed to generalize to T2D populations, and vice versa. Mixed-population studies provide some evidence of broader applicability, but adequately powered, diabetes-type-specific external validation remains necessary before models can be considered broadly generalizable.

The most important finding from a clinical perspective is that evidence for actual clinical benefit remains much less mature than evidence for prediction accuracy. Many included studies reported AUROC, RMSE, mean absolute error (MAE), mean absolute relative difference (MARD), F1-score, or related metrics, but considerably fewer demonstrated that these improvements changed TIR, hemoglobin A1c (HbA1c), hypoglycemia burden, or other clinically meaningful outcomes. This distinction was particularly evident because most studies evaluated model development or validation rather than prospective clinical implementation.

This distinction is critical. A model may accurately predict a hypoglycemic event without preventing that event. Clinical benefit requires an additional pathway: the prediction must be delivered at an appropriate time, interpreted correctly, followed by an actionable recommendation, and subsequently acted upon by the patient or healthcare professional. Therefore, the clinical effectiveness of AI should be conceptualized as a chain from data acquisition to prediction, decision support, intervention, behavioral or therapeutic response, and ultimately improved glycemic outcome.

Parcerisas et al. provide an example of this intermediate stage, using prediction-based decision strategies to simulate the potential mitigation of nocturnal hypoglycemia [35]. Their findings suggest that prediction can potentially be converted into preventive action, but simulation-based effectiveness should not be interpreted as equivalent to prospectively observed clinical benefit.

The strongest real-world evidence among the included studies came from Kannenberg et al. [2]. Their predictive-algorithm-driven digital therapeutic was evaluated in 118 adults with non-insulin-treated type 2 diabetes. After three months, HbA1c decreased by 0.67 percentage points overall and by 1.08 percentage points among participants with baseline HbA1c ≥7%; the proportion achieving HbA1c <7% increased from 38% to 60% [2]. These findings are clinically encouraging because they demonstrate that AI-supported personalization can potentially affect outcomes beyond prediction accuracy. However, this evidence represents a relatively small subset of the overall literature and should not be generalized to all AI-based prediction systems or to other diabetes populations without further validation.

The randomized Daily Dose study provides an important complementary example of translating prediction into an intervention [11]. The system used an evidential neural network to forecast nocturnal low-glucose risk and subsequently provided personalized bedtime snack recommendations. Although it did not significantly reduce nights with glucose <70 mg/dL, it significantly reduced more severe nocturnal events <54 mg/dL [11]. This illustrates both the potential and the complexity of AI-driven intervention: an intervention may produce meaningful benefits for a specific clinically important outcome without improving every glycemic metric.

The relatively limited evidence directly linking AI intervention to TIR is particularly noteworthy. TIR provides an integrated representation of glycemic exposure and is increasingly recognized as a clinically meaningful CGM-derived endpoint. Yet many AI studies remain focused on technical prediction metrics rather than demonstrating whether improved forecasting increases TIR or decreases time below range.

Future studies should therefore establish a clearer relationship between model performance and patient outcomes. Reporting only an improvement in AUROC or RMSE does not establish clinical utility. A clinically relevant evaluation should determine whether AI-assisted prediction leads to increased TIR, reduced time below range, fewer clinically significant hypoglycemic episodes, improved HbA1c, or reduced treatment burden. These outcomes should be evaluated prospectively and, where feasible, against appropriate usual-care or active-control comparators rather than inferred from model performance alone.

External validation remains another critical issue. Shao et al. specifically examined generalization of hypoglycemia prediction across independent populations and highlighted the challenges associated with transferring models between populations [9]. This issue is fundamental because CGM devices, treatment patterns, demographic characteristics, behavioral patterns, and disease phenotypes can differ substantially between datasets.

The same concern is evident in the increasingly sophisticated multimodal studies. Alkhateeb et al. emphasized patient-wise, leak-free evaluation and cross-phase validation [18], while Luo et al. incorporated held-out and temporal validation and testing on unseen patients [31]. These approaches are preferable to random record-level splitting because they provide a more realistic estimate of how a model may perform when deployed in new individuals.

Beyond external validation, future studies should also prioritize transparent reporting, calibration, clinically meaningful decision thresholds, and evaluation across relevant diabetes subgroups. Model discrimination alone may be insufficient for clinical implementation if predicted risks are poorly calibrated or if the selected threshold produces excessive false alerts or clinically inappropriate recommendations. Prospective evaluation should therefore assess not only whether a model predicts accurately but also whether its predictions improve decision-making without increasing treatment burden, alert fatigue, or unintended consequences.

Overall, the evidence indicates that the field is transitioning from proof-of-concept prediction toward personalized AI-enabled glycemic decision support. However, this transition remains incomplete, particularly because technical evidence substantially outweighs prospective evidence of clinical effectiveness. The next methodological priority should therefore be prospective validation in representative populations and direct evaluation of whether AI predictions improve clinically meaningful outcomes. In particular, TIR, time below range, hypoglycemia burden, HbA1c, and patient-reported treatment burden should become central endpoints alongside conventional AI performance metrics. Studies should also report diabetes type explicitly and evaluate whether model performance and clinical effectiveness are consistent across T1D, T2D, and mixed populations. Establishing this evidence will be essential for determining whether increasingly sophisticated AI systems can move beyond accurate prediction to safe, effective, and generalizable diabetes care.

Limitations

This review has several limitations. First, substantial heterogeneity across AI architectures, input variables, prediction horizons, outcome definitions, datasets, and performance metrics limited direct comparison and precluded meaningful quantitative pooling of predictive performance. Second, the evidence base was predominantly composed of prediction-model studies, with substantially fewer studies evaluating prospective clinical effectiveness or patient-centered outcomes such as TIR, HbA1c, hypoglycemia burden, or treatment burden. Third, the included studies were not evenly distributed across diabetes types, with a predominance of T1D populations and fewer studies specifically evaluating T2D or mixed T1D/T2D populations; therefore, the generalizability of reported findings across diabetes types remains uncertain. Fourth, many models were developed and evaluated using single-center, relatively small, or otherwise limited datasets, with inconsistent external validation, potentially restricting generalizability across populations, CGM devices, healthcare settings, and treatment regimens. Fifth, differences in study design and methodological quality introduced uncertainty regarding the strength of the evidence, with several studies demonstrating important risk-of-bias concerns, particularly regarding participant selection, sample size, validation strategies, and analysis. Finally, the rapidly evolving nature of AI technology means that newer architectures, multimodal approaches, foundation models, and validation strategies may not be fully represented in the currently available literature. These limitations should be considered when interpreting the promising predictive performance reported across the included studies and reinforce the need for prospective, externally validated, diabetes-type-specific studies using standardized and clinically meaningful outcomes.

## Conclusions

AI has demonstrated considerable potential for improving glucose prediction and supporting personalized glycemic management in adults with diabetes. Across the included studies, AI-based models showed promising performance for short-term glucose forecasting and prediction of clinically important glycemic events, particularly hypoglycemia and hyperglycemia, with increasing integration of multimodal data and individualized modeling approaches. However, the evidence base was predominantly derived from T1D populations, with fewer studies specifically evaluating T2D or mixed diabetes populations, which limits certainty regarding generalizability across diabetes types. Moreover, the evidence remains considerably stronger for technical predictive performance than for demonstrated clinical effectiveness. Direct evidence that AI-driven interventions improve clinically meaningful outcomes, particularly time in range, remains limited. Future research should therefore move beyond model development and internal validation toward prospective, externally validated, and, where appropriate, randomized studies that evaluate whether AI-supported predictions translate into improved patient outcomes. Standardized reporting of TIR, time below range, HbA1c, clinically significant hypoglycemic events, treatment burden, and other patient-centered outcomes should be prioritized, alongside transparent reporting of calibration and performance across relevant diabetes populations. Overall, AI represents a promising component of personalized diabetes care, but stronger evidence of clinical effectiveness, generalizability, and safety is required before widespread implementation can be confidently recommended.

## Appendices

Appendix 1. Search strategies

PubMed Search String

("Diabetes Mellitus"[Mesh] OR "Diabetes Mellitus, Type 1"[Mesh] OR "Diabetes Mellitus, Type 2"[Mesh] OR diabet*[tiab] OR "type 1 diabetes"[tiab] OR "type 2 diabetes"[tiab] OR T1DM[tiab] OR T2DM[tiab]) AND ("Artificial Intelligence"[Mesh] OR "Machine Learning"[Mesh] OR "Deep Learning"[tiab] OR "artificial intelligence"[tiab] OR "machine learning"[tiab] OR "machine-learning"[tiab] OR "deep learning"[tiab] OR "neural network*"[tiab] OR "artificial neural network*"[tiab] OR "recurrent neural network*"[tiab] OR LSTM[tiab] OR "long short-term memory"[tiab] OR transformer*[tiab] OR "random forest*"[tiab] OR XGBoost[tiab] OR "gradient boosting"[tiab] OR "support vector machine*"[tiab] OR "reinforcement learning"[tiab] OR "ensemble learning"[tiab] OR "predictive algorithm*"[tiab] OR "prediction algorithm*"[tiab] OR "predictive model*"[tiab] OR "predictive analytics"[tiab]) AND ("Continuous Glucose Monitoring"[Mesh] OR "Blood Glucose Self-Monitoring"[Mesh] OR "continuous glucose monitoring"[tiab] OR "continuous glucose monitor*"[tiab] OR CGM[tiab] OR rtCGM[tiab] OR "real-time glucose monitoring"[tiab] OR "real time glucose monitoring"[tiab] OR "intermittently scanned glucose monitoring"[tiab] OR "flash glucose monitoring"[tiab] OR "glucose sensor*"[tiab] OR "glucose monitoring"[tiab] OR "interstitial glucose"[tiab]) AND ("glucose prediction"[tiab] OR "glucose forecasting"[tiab] OR "glucose forecast*"[tiab] OR "glycemic prediction"[tiab] OR "glycaemic prediction"[tiab] OR "glucose trajectory"[tiab] OR "glucose excursion*"[tiab] OR "hypoglycemia prediction"[tiab] OR "hypoglycaemia prediction"[tiab] OR "hyperglycemia prediction"[tiab] OR "hyperglycaemia prediction"[tiab] OR "hypoglycemia alert*"[tiab] OR "hypoglycaemia alert*"[tiab] OR "hyperglycemia alert*"[tiab] OR "hyperglycaemia alert*"[tiab] OR "predictive alert*"[tiab] OR "predictive alarm*"[tiab] OR "early warning"[tiab] OR "risk prediction"[tiab] OR "glycemic risk"[tiab] OR "glycaemic risk"[tiab] OR "decision support"[tiab] OR "clinical decision support"[tiab] OR "personalized management"[tiab] OR "personalised management"[tiab] OR "personalized intervention*"[tiab] OR "personalised intervention*"[tiab] OR "digital therapeutic*"[tiab] OR "automated insulin delivery"[tiab] OR "closed-loop"[tiab] OR "closed loop"[tiab])

Scopus Search String

TITLE-ABS-KEY((diabet* OR "type 1 diabetes" OR "type 2 diabetes" OR T1DM OR T2DM OR "diabetes mellitus") AND ("artificial intelligence" OR "machine learning" OR "machine-learning" OR "deep learning" OR "neural network*" OR "artificial neural network*" OR "recurrent neural network*" OR LSTM OR "long short-term memory" OR transformer* OR "random forest*" OR XGBoost OR "gradient boosting" OR "support vector machine*" OR "reinforcement learning" OR "ensemble learning" OR "predictive algorithm*" OR "prediction algorithm*" OR "predictive model*" OR "predictive analytics") AND ("continuous glucose monitoring" OR "continuous glucose monitor*" OR CGM OR rtCGM OR "real-time glucose monitoring" OR "real time glucose monitoring" OR "intermittently scanned glucose monitoring" OR "flash glucose monitoring" OR "glucose sensor*" OR "glucose monitoring" OR "interstitial glucose") AND ("glucose prediction" OR "glucose forecasting" OR "glucose forecast*" OR "glycemic prediction" OR "glycaemic prediction" OR "glucose trajectory" OR "glucose excursion*" OR "hypoglycemia prediction" OR "hypoglycaemia prediction" OR "hyperglycemia prediction" OR "hyperglycaemia prediction" OR "hypoglycemia alert*" OR "hypoglycaemia alert*" OR "hyperglycemia alert*" OR "hyperglycaemia alert*" OR "predictive alert*" OR "predictive alarm*" OR "early warning" OR "risk prediction" OR "glycemic risk" OR "glycaemic risk" OR "decision support" OR "clinical decision support" OR "personalized management" OR "personalised management" OR "personalized intervention*" OR "personalised intervention*" OR "digital therapeutic*" OR "automated insulin delivery" OR "closed-loop" OR "closed loop"))

Web of Science Core Collection

TS=((diabet* OR "type 1 diabetes" OR "type 2 diabetes" OR T1DM OR T2DM OR "diabetes mellitus") AND ("artificial intelligence" OR "machine learning" OR "machine-learning" OR "deep learning" OR "neural network*" OR "artificial neural network*" OR "recurrent neural network*" OR LSTM OR "long short-term memory" OR transformer* OR "random forest*" OR XGBoost OR "gradient boosting" OR "support vector machine*" OR "reinforcement learning" OR "ensemble learning" OR "predictive algorithm*" OR "prediction algorithm*" OR "predictive model*" OR "predictive analytics") AND ("continuous glucose monitoring" OR "continuous glucose monitor*" OR CGM OR rtCGM OR "real-time glucose monitoring" OR "real time glucose monitoring" OR "intermittently scanned glucose monitoring" OR "flash glucose monitoring" OR "glucose sensor*" OR "glucose monitoring" OR "interstitial glucose") AND ("glucose prediction" OR "glucose forecasting" OR "glucose forecast*" OR "glycemic prediction" OR "glycaemic prediction" OR "glucose trajectory" OR "glucose excursion*" OR "hypoglycemia prediction" OR "hypoglycaemia prediction" OR "hyperglycemia prediction" OR "hyperglycaemia prediction" OR "hypoglycemia alert*" OR "hypoglycaemia alert*" OR "hyperglycemia alert*" OR "hyperglycaemia alert*" OR "predictive alert*" OR "predictive alarm*" OR "early warning" OR "risk prediction" OR "glycemic risk" OR "glycaemic risk" OR "decision support" OR "clinical decision support" OR "personalized management" OR "personalised management" OR "personalized intervention*" OR "personalised intervention*" OR "digital therapeutic*" OR "automated insulin delivery" OR "closed-loop" OR "closed loop"))

Appendix 2. Risk of bias assessment of AI-based prediction models

**Table 4:** Signaling-question assessment of risk of bias in the development of AI-based prediction models using PROBAST+AI Y, yes; PY, probably yes; PN, probably no; NI, no information; RoB, risk of bias; PROBAST+AI, Prediction Model Risk Of Bias ASsessment Tool Plus Artificial Intelligence.

Study	Participants/data	Predictors	Outcome	Analysis	Overall RoB
Alkhateeb et al., 2026 [18]	PY	Y	Y	PN	High
Alvarado et al., 2023 [19]	PN	Y	Y	PN	High
Amar et al., 2020 [20]	PN	Y	Y	PN	High
Brügger et al., 2025 [1]	PN	Y	Y	PN	High
Cichosz et al., 2021a [21]	Y	Y	Y	Y	Low
Cichosz et al., 2021b [22]	Y	Y	Y	Y	Low
Cichosz et al., 2024 [23]	Y	Y	Y	Y	Low
Gong et al., 2024 [8]	PN	Y	Y	Y	High
Herrero et al., 2024 [24]	Y	Y	Y	Y	Low
Khadem et al., 2026 [25]	PN	Y	Y	PN	High
Kim et al., 2022 [26]	PN	Y	Y	PN	High
Kladov et al., 2024 [27]	Y	Y	Y	PN	High
Kozinetz et al., 2024 [28]	Y	Y	Y	Y	Low
Kushner et al., 2020 [29]	PN	Y	Y	PN	High
Lara-Abelenda et al., 2025 [30]	Y	Y	Y	PN	High
Luo et al., 2025 [31]	Y	Y	Y	Y	Low
Mosquera-Lopez et al., 2019 [32]	PN	Y	Y	Y	High
Mosquera-Lopez & Jacobs, 2022 [33]	Y	Y	Y	Y	Low
Mosquera-Lopez et al., 2023 [34]	Y	Y	Y	Y	Low
Parcerisas et al., 2022 [35]	PN	Y	Y	PN	High
Rabby et al., 2021 [36]	PN	Y	Y	PN	High
Rodríguez-Almeida et al., 2025 [37]	PN	Y	Y	PN	High
Shao et al., 2024 [9]	Y	Y	Y	Y	Low
Shilo et al., 2022 [38]	Y	Y	Y	PN	High
van Doorn et al., 2021 [7]	Y	Y	Y	Y	Low
Zhu et al., 2022a [39]	PN	Y	Y	PN	High
Zhu et al., 2022b [40]	Y	Y	Y	PN	High
Zhu et al., 2026 [41]	Y	Y	Y	Y	Low

**Table 5:** Risk-of-bias assessment for evaluation and validation of AI-based prediction models using PROBAST+AI PROBAST+AI, Prediction Model Risk Of Bias ASsessment Tool Plus Artificial Intelligence.

Study	Participants/data	Predictors	Outcome	Analysis/evaluation	Overall RoB
Alkhateeb et al., 2026 [18]	High	Low	Low	High	High
Alvarado et al., 2023 [19]	High	Low	Low	High	High
Amar et al., 2020 [20]	High	Low	Low	High	High
Brügger et al., 2025 [1]	High	Low	Low	High	High
Cichosz et al., 2021a [21]	Low	Low	Low	Low	Low
Cichosz et al., 2021b [22]	Low	Low	Low	Low	Low
Cichosz et al., 2024 [23]	Low	Low	Low	Low	Low
Gong et al., 2024 [8]	High	Low	Low	Low	High
Herrero et al., 2024 [24]	Low	Low	Low	Low	Low
Khadem et al., 2026 [25]	High	Low	Low	High	High
Kim et al., 2022 [26]	High	Low	Low	High	High
Kladov et al., 2024 [27]	Low	Low	Low	High	High
Kozinetz et al., 2024 [28]	Low	Low	Low	Low	Low
Kushner et al., 2020 [29]	High	Low	Low	High	High
Lara-Abelenda et al., 2025 [30]	Low	Low	Low	High	High
Luo et al., 2025 [31]	Low	Low	Low	Low	Low
Mosquera-Lopez et al., 2019 [32]	High	Low	Low	Low	High
Mosquera-Lopez & Jacobs, 2022 [33]	Low	Low	Low	Low	Low
Mosquera-Lopez et al., 2023 [34]	Low	Low	Low	Low	Low
Parcerisas et al., 2022 [35]	High	Low	Low	High	High
Rabby et al., 2021 [36]	High	Low	Low	High	High
Rodríguez-Almeida et al., 2025 [37]	High	Low	Low	High	High
Shao et al., 2024 [9]	Low	Low	Low	Low	Low
Shilo et al., 2022 [38]	Low	Low	Low	High	High
van Doorn et al., 2021 [7]	Low	Low	Low	Low	Low
Zhu et al., 2022a [39]	High	Low	Low	High	High
Zhu et al., 2022b [40]	Low	Low	Low	High	High
Zhu et al., 2026 [41]	Low	Low	Low	Low	Low

**Table 6:** Overall risk of bias and applicability of AI-based prediction models according to PROBAST+AI

Study	Overall risk of bias	Participants/data applicability	Predictor applicability	Outcome applicability	Overall applicability
Alkhateeb et al., 2026 [18]	High	Low	Low	Low	Low
Alvarado et al., 2023 [19]	High	Low	Low	Low	Low
Amar et al., 2020 [20]	High	High	Low	Low	High
Brügger et al., 2025 [1]	High	High	Low	Low	High
Cichosz et al., 2021a [21]	Low	Low	Low	Low	Low
Cichosz et al., 2021b [22]	Low	Low	Low	Low	Low
Cichosz et al., 2024 [23]	Low	Low	Low	Low	Low
Gong et al., 2024 [8]	High	High	Low	Low	High
Herrero et al., 2024 [24]	Low	Low	Low	Low	Low
Khadem et al., 2026 [25]	High	Low	Low	Low	Low
Kim et al., 2022 [26]	High	High	Low	Low	High
Kladov et al., 2024 [27]	High	Low	Low	Low	Low
Kozinetz et al., 2024 [28]	Low	Low	Low	Low	Low
Kushner et al., 2020 [29]	High	Low	Low	Low	Low
Lara-Abelenda et al., 2025 [30]	High	Low	Low	Low	Low
Luo et al., 2025 [31]	Low	Low	Low	Low	Low
Mosquera-Lopez et al., 2019 [32]	High	High	Low	Low	High
Mosquera-Lopez & Jacobs, 2022 [33]	Low	Low	Low	Low	Low
Mosquera-Lopez et al., 2023 [34]	Low	Low	Low	Low	Low
Parcerisas et al., 2022 [35]	High	High	Low	High	High
Rabby et al., 2021 [36]	High	Low	Low	Low	Low
Rodríguez-Almeida et al., 2025 [37]	High	Low	Low	Low	Low
Shao et al., 2024 [9]	Low	Low	Low	Low	Low
Shilo et al., 2022 [38]	High	Low	Low	Low	Low
van Doorn et al., 2021 [7]	Low	High	Low	Low	High
Zhu et al., 2022a [39]	High	Low	Low	Low	Low
Zhu et al., 2022b [40]	High	Low	Low	Low	Low
Zhu et al., 2026 [41]	Low	Low	Low	Low	Low

Appendix 3. Risk of bias assessment of the randomized clinical intervention

**Table 7:** Risk of bias assessment of randomized clinical intervention evaluating AI-driven glycemic decision support using the Cochrane Risk of Bias 2 (RoB 2) tool PROBAST+AI, Prediction Model Risk Of Bias ASsessment Tool Plus Artificial Intelligence.

Study	D1: Randomization process	D2: Deviations from intended interventions	D3: Missing outcome data	D4: Measurement of outcome	D5: Selection of reported result	Overall
Mosquera-Lopez et al., 2025 [11] (DailyDose)	Some concerns	Low	Low	Low	Low	Some concerns

Appendix 4. Risk of bias assessment of the non-randomized intervention-effect study

**Table 8:** Risk of bias assessment of the non-randomized intervention-effect study using ROBINS-I ROBINS-I: Risk Of Bias In Non-randomized Studies of Interventions.

Study	D1: Bias due to confounding	D2: Bias in selection of participants	D3: Bias in classification of interventions	D4: Bias due to deviations from intended interventions	D5: Bias due to missing data	D6: Bias in measurement of outcomes	D7: Bias in selection of reported result	Overall
Kannenberg et al., 2026 [2]	Serious	Moderate	Low	Moderate	Low	Low	Moderate	Serious
